# Electroacupuncture and conventional drugs treatment combination improved quality of life in patients with heart failure reduced ejection fraction: a single-blinded randomized controlled trial

**DOI:** 10.1186/s43044-025-00698-0

**Published:** 2025-10-29

**Authors:** Dwi Surya Supriyana, Tonang Dwi Ardyanto, Ida Nurwati, Didik Gunawan Tamtomo

**Affiliations:** 1 Doctoral Program of Medical Sciences, Faculty of Medicine, Universitas Sebelas Maret, Surakarta, Indonesia; 2 Universitas Sebelas Maret (UNS) Teaching Hospital, Surakarta, Indonesia; 3 Department of Clinical Pathology, Faculty of Medicine, Universitas Sebelas Maret, Surakarta, Indonesia; 4 Department of Biochemistry, Faculty of Medicine Universitas Sebelas Maret, Surakarta, Indonesia

**Keywords:** Acupuncture, Electrostimulation, Heart failure, Ejection fraction, Functional capacity, Quality of life

## Abstract

**Background:**

Heart failure (HF) is a major cardiovascular disease (CVD) with high morbidity and mortality. Research on the effect of electroacupuncture (EA) on the quality of life (QoL) of HF patients with reduced ejection fraction (HFrEF) remains limited. This study aims to determine the role of combining EA with conventional treatment in improving QoL for patients with HFrEF.

**Methods:**

This single-blind, randomized controlled trial employed a pre- and post-test design at the Heart Failure and Acupuncture Clinics of UNS Hospital, Indonesia. Thirty-four participants participated, with random assignment to either an intervention or a control group. While the intervention group received pharmacological therapy combined with electroacupuncture, the control group received only pharmacological therapy. All pharmacological treatments were administered according to cardiologists' prescriptions in alignment with the 2021 European Society of Cardiology guidelines. In the intervention group, participants underwent 32 EA sessions conducted by a medical acupuncture specialist, delivered twice weekly for 30 min per session. The primary outcomes were: (1) Change in Left Ventricular Ejection Fraction (LVEF); (2) Change in six-minute walk distance; and (3) Change in Kansas City Cardiomyopathy Questionnaire-23 (KCCQ-23) score, measured before and after the intervention period.

**Results:**

The combination of pharmacological therapy and EA produced a significant increase in the mean of LVEF by 14.07 ± 5.67%, an improvement in mean 6MWD by 348.82 ± 61.23 m, and an increase in mean KCCQ-23 score to 34.94 ± 5.99. Statistically significant differences were observed between the intervention and control groups for all measured outcomes (*p* < 0.05).

**Conclusions:**

The combination of conventional treatment and EA significantly improves quality of life in patients with HFrEF, representing a promising adjuvant therapy for heart failure.

## Introduction

Heart failure (HF) is a clinical syndrome characterized by impaired cardiac muscle function resulting in inadequate systemic perfusion, with high morbidity and mortality [1]. Approximately 38 million individuals are affected globally, including ten million in Indonesia [2]. Management of heart failure with reduced ejection fraction (HFrEF) has advanced with updates of guideline-directed medical therapy, such as evidence-based pharmacological agents and device therapies, as recommended by the 2021 European Society of Cardiology (ESC) and American College of Cardiology/American Heart Association (ACC/AHA) guidelines [3]. Despite this, mortality and readmission rates remain high, emphasizing the need for optimized management. Addressing comorbidities, system inefficiencies, and individualized patient factors is imperative [2].

Ejection fraction (EF) is a critical prognostic indicator in HF [4, 5]. Heart Failure (HF) is classified according to left ventricular ejection fraction (LVEF). Heart failure with reduced ejection fraction (HFrEF) is defined as an LVEF of 40% or less. HFrEF accounts for about half of all HF cases and typically requires hospitalization [3, 6]. Patients not only experience clinical symptoms such as dyspnea, fatigue, and peripheral edema, but also face psychological and mental health issues that further diminish quality of life (QoL) [7].

Acupuncture, especially electroacupuncture (EA), is a promising intervention for cardiovascular disease (CVD), including HF [8, 9]. EA in HF therapy works by improving neurohormonal reactions, which affect hemodynamic defense, inflammation, and ventricular remodelling [10]. Activating the Hypothalamic–Pituitary–Adrenal Axis (HPA Axis), a central neuroendocrine system, acupuncture helps maintain perfusion pressure and cardiac output (CO) [11]. EA also reduces Renal Sympathetic Nerve Activity (RSNA) and Cardiac Sympathetic Afferent Reflex (CSAR), thereby improving cardiac function, remodeling, baroreflex function, and hemodynamic parameters [12, 13].

Acupuncture research in HF has been conducted internationally, particularly in countries utilizing Traditional Chinese Medicine (TCM). Although multiple studies have examined its role in HF through various modalities, significant knowledge gaps persist. The potential application of acupuncture in heart failure with reduced ejection fraction (HFrEF) requires further investigation, especially within the Indonesian context, where research remains limited.

## Materials and methods

### Study design and setting

A single-blind randomized controlled trial (RCT) with a pretest–posttest design was conducted at the Heart Failure Clinic and Acupuncture Clinic of Universitas Sebelas Maret (UNS) Hospital, Central Java, Indonesia, between May and November 2024. The study was designed to detect significant changes in the Left Ventricular Ejection Fraction (LVEF) as the primary outcome. The research protocol complied with the Declaration of Helsinki. Ethical approval was granted, and the research background was submitted to the UNS hospital to obtain a research permit. All participants provided written informed consent.

### Study population and follow-up

We calculated the sample size using a test for differences between two independent proportions, accounting for a 20% dropout rate, with a 0.05 significance level, and 0.80 power. Seventeen patients were assigned to each group: the Intervention Group, which received pharmacological drugs with EA, and the Control Group, which received pharmacological drugs without EA. A research assistant prepared sealed and shuffled envelopes, each with a group code. Patients selected an envelope, unaware of their group. After all envelopes were assigned, the group codes were disclosed to the relevant study staff; only the principal investigator was aware of the allocations during the process. The cardiologist remained blinded, and participants were instructed not to reveal their group to echocardiography operators or other staff.

The participants were selected by reviewing medical records and screened for eligibility using the following inclusion criteria: aged ≥ 18 years; a confirmed diagnosis of stable HFrEF (NYHA class II-III, EF ≤ 40%) for more than three months, according to the 2021 Guidelines of the ESC [3]; and willing to participate in this study. The exclusion criteria for all participants were that they had recently undergone acupuncture therapy in the past three months and had a history of massive bleeding, malignancy, pregnancy, breastfeeding, severe skin damage, a fever over 38 °C, infection, inflammation, or the use of metal implantation devices.

All groups received standard cardiologist monitoring and conventional drug treatment, following the 2021 ESC guidelines for HF [3], and the national drug formulary of the Indonesian National Health Insurance. All participants in this study were patients covered by the Indonesian national health insurance. The intervention group received pharmacological drugs, along with electroacupuncture (EA) therapy, administered by a medical acupuncture specialist. The control group received pharmacological treatment without any form of acupuncture. Both groups underwent standard drug therapy for four months and were monitored monthly by a cardiologist to ensure adherence. The intervention group completed 32 sessions of EA during the study period. The selection of 32 sessions was based on evidence from previous studies indicating that extended electroacupuncture (EA) exposure optimizes therapeutic outcomes, especially improvements in cardiac function and patient quality of life. Left Ventricle Ejection Fraction (LVEF), Six-Minute Walking Distance (6MWD), and Kansas City Cardiomyopathy Questionnaire-23 (KCCQ-23) data were collected for all groups at both the pre-therapy (1st session) and post-therapy (32nd session) time points.

### Electroacupuncture therapy procedure

**Fig. 1:**
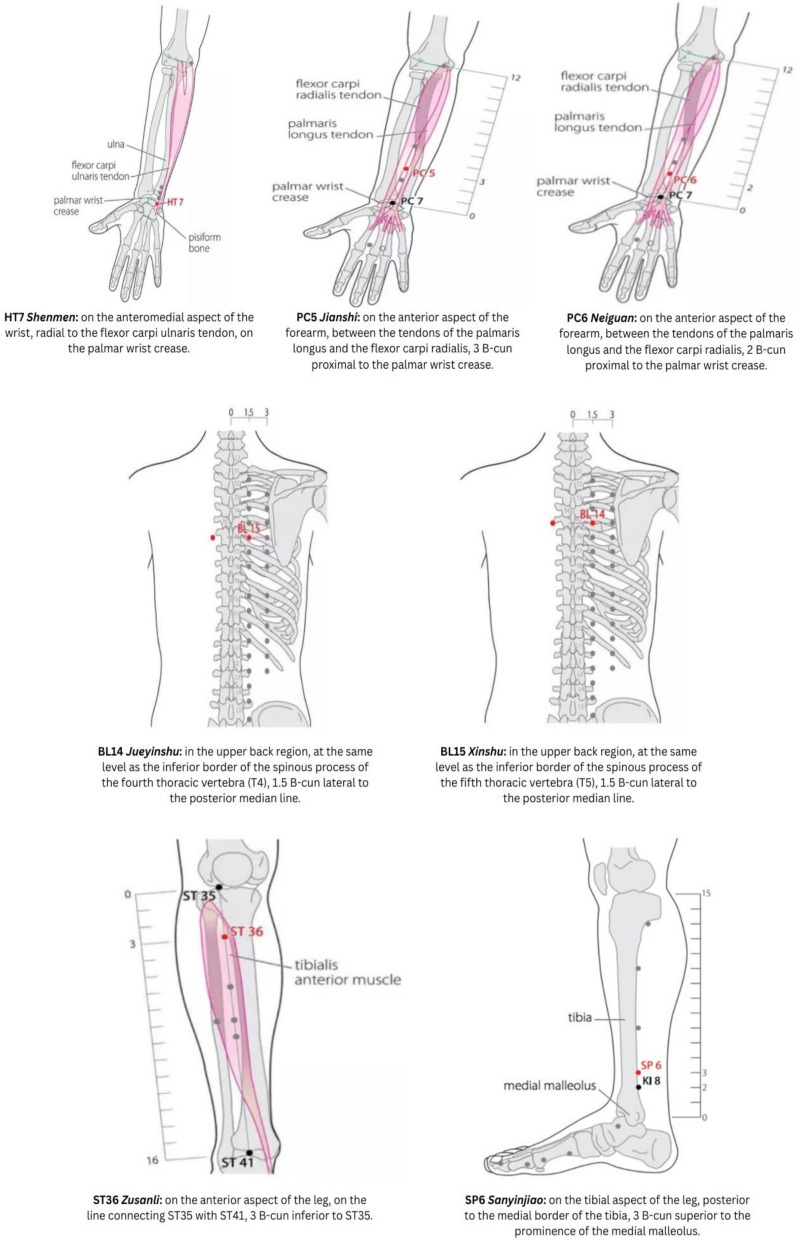
Location of points [35]

The electroacupuncture (EA) protocol comprised 32 sessions, conducted twice weekly for 30 min per session. Disposable, sterile stainless steel acupuncture needles (0.25 × 25 mm) were inserted bilaterally and perpendicularly. The patient was positioned supine for stimulation at bilateral HT7 (*Shenmen)*, PC5 (*Jianshi)*, PC6 (*Neiguan)*, ST36 (*Zusanli)*, and SP6 (Sanyinjiao) acupoints. Subsequently, the patient was repositioned in the prone position for stimulation at bilateral BL14 (Jueyinshu) and BL15 (Xinshu) acupoints. Acupuncture points were stimulated for 30 min using continuous low-intensity, low-frequency electrical currents (2 mA, 2–4 Hz) delivered by a Hwato-type SDZ-V 105 digital electrostimulation device (Fig. 1).

### Patient safety monitoring procedures for electroacupuncture treatment

Researchers conduct a thorough assessment before the procedure, use sterile and disposable needles to prevent infection, and ensure patient comfort throughout the process. They also monitor the patient's body responses, such as tingling or pain sensations, by actively communicating to address complaints or adverse events.

### Echocardiography measurements

Echocardiographic assessment was conducted by a single cardiologist. Left Ventricular Ejection Fraction (LVEF) was determined using a Philips EPIQ 7 C Ultrasound System (Koninklijke Philips N.V.) equipped with an XS1 probe. The biplanar Simpson method, recognized as the gold standard for echocardiographic imaging, was utilized to evaluate left ventricular function. This technique provides an accurate assessment of cardiac segmental motion abnormalities. LVEF was calculated using an apical four-chamber scanning approach, which enables visualization of all four cardiac chambers and focuses on the left ventricle for precise measurement [14].

### The six-minute walking test (6MWT) and KCCQ-23 measurements

All patients completed the 6-Minute Walk Test (6MWT) before and after the study, following standardized instructions and receiving encouragement. Participants were instructed to walk as quickly and as far as possible. If necessary, they could slow down or stop. Rest was permitted at any point before or during the test. The test was discontinued if the patient experienced chest pain, severe dyspnea, cramps, disorientation, diaphoresis, or pallor. The Six-Minute Walk Test (6MWT), which measures walking distance (6MWD), is commonly used in cardiovascular assessments to evaluate functional capacity. This test is considered efficient, well-tolerated, and more representative of the daily activities of patients with HF [15, 16].

The Kansas City Cardiomyopathy Questionnaire-23 (KCCQ-23) is a disease-specific, patient-reported outcome measure with strong psychometric properties, designed for individuals with HF. The KCCQ-23 correlates patients' subjective assessments of their HF condition with disease-specific health risks and quality of life (QoL) [17]. Patients completed the KCCQ-23 at the beginning and end of therapy. The questionnaire includes 23 items that evaluate physical condition, symptoms, self-efficacy, daily activity performance, social limitations, and QoL.

### Statistical analysis

Descriptive statistical analysis summarized the data distribution by calculating means, standard deviations, percentages, and frequencies. The Shapiro–Wilk test was then used to assess the normality of the data. To provide clarity regarding the hierarchy of outcomes, tests have been organized from primary to exploratory with appropriate alpha control. Our primary outcomes, mainly focused on LVEF, were analyzed using paired or independent t-tests when normality assumptions were met, ensuring confirmatory findings. Secondary and exploratory outcomes were assessed using non-parametric tests, such as the Mann–Whitney test and the Wilcoxon signed rank test, when data did not conform to normality assumptions due to small sample sizes or skewed distributions. This pre-specified plan ensures that the choice of statistical test aligns with data characteristics and distinguishes between confirmatory and hypothesis-generating results. Statistical significance was defined as a p-value less than 0.05.

## Results

### Demographics and baseline characteristics

A total of 34 patients with HFrEF were included, with males comprising 61.8% and females the remainder. The participants had a median age of 54.67 years, and a mean age of 55.68 ± 12.13 years; their baseline mean LVEF was 27.12 ± 9.19. Most participants (73.53%) had comorbid conditions. Further sociodemographic and clinical characteristics are presented in Table 1 below:

**Table 1:** Characteristics of the study participants with HFrEF (N = 34)

Characteristics	Group		*p*-value
	Intervention (Drugs with Acupuncture) (n = 17)	Control (Drugs without Acupuncture) (n = 17)	
Demographics and Comorbidities			
Age (years)			
mean ± SD	52 ± 13.59	59.35 ± 9.48	0.08
< 46	4 (11.8)	1 (2.8)	
46–64	11 (32.4)	11 (32.4)	
> 64	2 (5.9)	5 (14.7)	
Gender, n (%)			
Male	11 (64.71)	10 (58.82)	
Female	6 (35.29)	7 (41.18)	0.66
BMI (kg/m^2^)	22.37 ± 4.22	24.99 ± 4.35	0.17
Diabetes mellitus Type 2, n (%)	3 (17.65)	5 (29.41)	0.41
Hypertension, n (%)	6 (35.29)	9 (52.94)	0.32
COPD, n (%)	1 (5.88)	1 (5.88)	1.00
Smoking history, n (%)	4 (23.53)	5 (29.41)	0.74
Physical Examination and Laboratories	25.58 ± 8.89	28.67 ± 9.49	
Mean HR (beats/min)	75.41 ± 9.02	79.06 ± 20.89	0.39
Mean MAP	90.04 ± 11.84	91.24 ± 18.41	0.81
BP (mmHg)			
Systolic BP (mean ± SD)	120.71 ± 19.28	123.47 ± 24.46	0.72
Diastolic BP (mean ± SD)	74.71 ± 9.51	75.12 ± 16.03	0.94
Oximetry (SpO2), %	97.82 ± 1.7	98.29 ± 1.0	0.34
Glucose ad random (mg/dL)	102.47 ± 25.48	110.59 ± 50,68	0.22
Haemoglobin (mg/dL)	13.88 ± 1.23	13.84 ± 1.35	0.95
Creatinine (mg/dL)	1.26 ± 0.5	1.22 ± 0.53	0.52
Echocardiography			
Baseline LVEF (mean ± SD)	25.58 ± 8.89	28.67 ± 9.49	0.66
Conventional Drugs Treatment based on 2021 ESC Guidelines for HF in National Formulary Drugs of the Indonesian National Health Insurance, n (%)			
ACE-I	8 (47.06)	8 (47.06)	0.48
Betablockers	12 (70.59)	13 (76.47)	0.71
ARBs	7 (41.18)	8 (47.06)	0.71
CCB	3 (17.65)	3 (17.65)	1.00
Statin	10 (58.82)	11 (64.71)	0.26
Diuretic	6 (17.4)	3 (8.7)	
Furosemide	10 (58.82)	11 (64.71)	0.71
Spironolactone	10 (58.82)	11 (64.71)	0.66
Antiplatelet (Clopidogrel/Aspirin)	10 (58.82)	8 (47.06)	0.48
Anticoagulant (Warfarin)	8 (47.06)	5 (29.41)	0.26
Metformin	2 (11.76)	3 (17.65)	0.66

### Result of Left Ventricle Ejection Fraction (LVEF) measurements analysis

In the Intervention Group, the left ventricular ejection fraction (LVEF) increased by an average of 14.07 ± 5.67%, a statistically significant change (*p* < 0.001). In contrast, the Control Group exhibited a mean LVEF increase of 6.22 ± 14.45%, which was not statistically significant (*p* = 0.095). The difference in the change in LVEF between the two groups was also statistically significant (*p* = 0.04; see Table 2).

**Table 2:** Results of Test Analysis Pre- and Post-Therapy on LVEF Measurement

LVEF value	Group		*p*-value
	Intervention Group (mean ± SD)	Control Group (mean ± SD)	
Pre-therapy	25.58 ± 8.89	28.67 ± 9.49	0.34^a^
Post-therapy	39.32 ± 11.19	34.89 ± 13.72	0.31^a^
p-value (post–pre)	< 0.001^b^	0.095^b^	
Delta (Δ)	14.07 ± 5.67	6.22 ± 14.45	0.04^a^

Note: Observation results are described using mean ± standard deviation (SD)

^a^Unpaired group difference test passed normality requirements (independent t-test);

^b^Pairwise group difference test passed normality requirements (paired t-test)

### Results of 6MWD measurement analysis

In the Intervention groups, patients receiving combination pharmacological drug therapy with EA showed an increase in 6MWD by a mean of 348.82 ± 91.23 m, representing a significant change (*p* < 0.001) over 32 sessions. The Control Group, which received standard treatment without EA, achieved a lower mean increase of 97.06 ± 80.68 m, with a significant group difference (*p* < 0.001). Table 3 provides detailed 6MWD results:

**Table 3:** Results of Test Analysis Pre- and Post-Therapy on 6MWD Measurements

6MWD value	Group		*p*-value
	Intervention Group (mean ± SD)	Control Group (mean ± SD)	
Pre-therapy	361.18 ± 115.59	312.35 ± 160.96	0.18^a^
Post-therapy	710.00 ± 138.97	409.41 ± 215.65	< 0.001^b^
p-value (post–pre)	< 0.001^c^	< 0.001^d^	
Delta (Δ)	348.82 ± 61.23	97.06 ± 80.68	< 0.001^b^

Note: Observation results are described using mean ± standard deviation (SD)

^a^Unpaired group difference test did not meet the requirements for normality (Mann–Whitney test);

^b^Unpaired group difference test passed normality requirements (independent t-test);

^c^Pairwise group difference test passed normality requirements (paired t-test)

^d^The paired group difference test did not meet the requirements for normality (Wilcoxon test);

### Results of the Kansas City Cardiomyopathy Questionnaire-23 (KCCQ-23) measurement analysis

The Intervention Group demonstrated an increase in the average KCCQ-23 score from 44.29 ± 3.8 to 79.18 ± 8.9 (*p* < 0.001), while the Control Group also showed an increase from 44.88 ± 5.5 to 55.47 ± 12.4 (*p* < 0.001). The KCCQ-23 delta score change was greater in the Intervention Group compared to the Control Group (34.94 ± 5.99 vs. 9.71 ± 8.07; *p* < 0.001). These data indicate a larger improvement with the combination of a pharmacological drug with EA (Table 4).

**Table 4:** Results of Test Analysis Pre- and Post-Therapy on KCCQ-23 Measurements

KCCQ-23 value	Group		*p*-value
	Intervention Group (mean ± SD)	Control Group (mean ± SD)	
Pre-therapy	44.29 ± 3.84	44.88 ± 5.48	0.89^a^
Post-therapy	79.18 ± 8.9	55.47 ± 12.39	< 0.001^a^
p-value (post–pre)	< 0.001^b^	< 0.001^b^	
Delta (Δ)	34.94 ± 5.99	9.71 ± 8.07	0.001^c^

Note: Observation results are described using mean ± SD

^a^Unpaired group difference test does not pass normality requirements (Mann–Whitney test);

^b^Pairwise group difference test does not pass normality requirements (Wilcoxon signed rank test);

^c^Unpaired group difference test passed normality requirements (independent t-test)

**Fig. 2:**
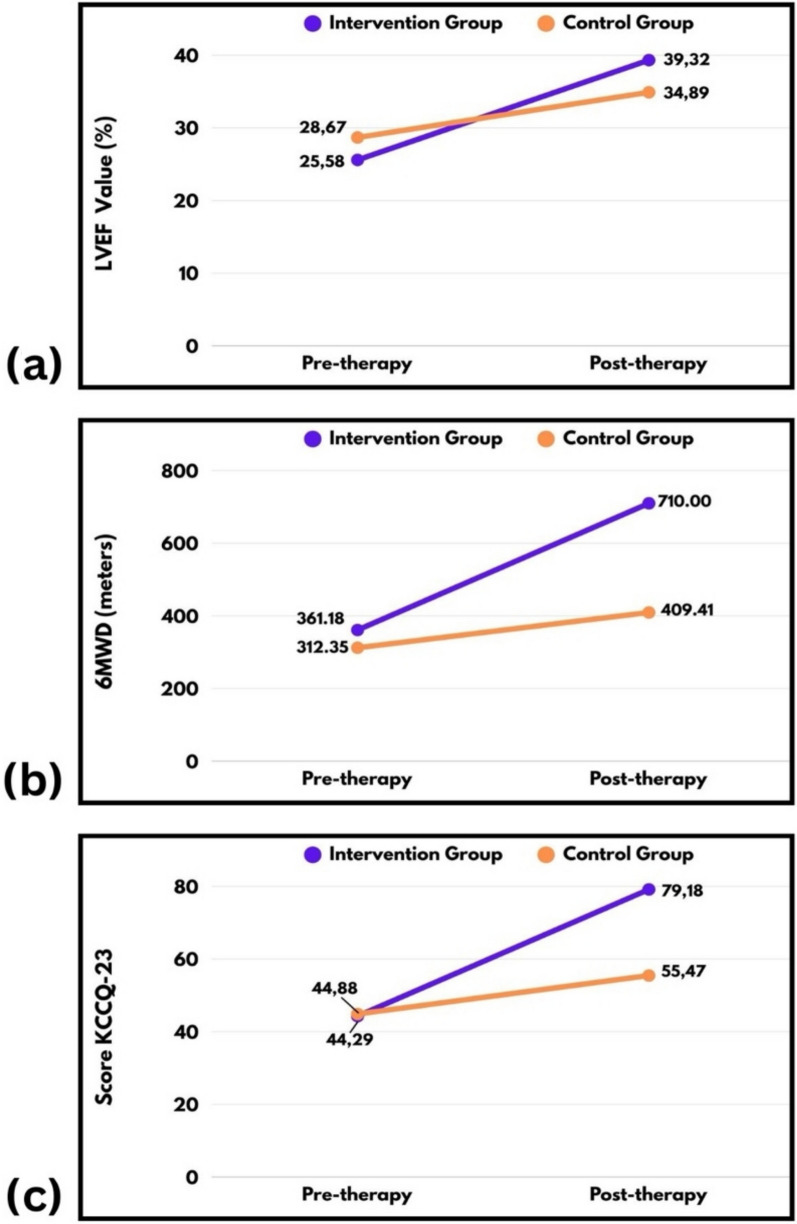
Trends for LVEF, 6MWD, and KCCQ-23 Measurement in the Two Study Groups. **a** Trends for LVEF Measurement in the Two Study Groups. **b** Trends for 6MWD Measurement in the Two Study Groups. **c** Trends for KCCQ-23 Measurement in the Two Study Groups

The measurement results of each research variable are shown in Fig. 2 below:

## Discussion

Heart failure (HF) is considered the last stage of HF and the cause of morbidity and mortality worldwide [2]. It is characterized by overactivation of the neurohormonal system, endothelial dysfunction, physical exercise intolerance, high mortality rates, and poor QoL [7]. Coronary Artery Disease (CAD), the primary cause of HFrEF, plays a significant role by leading to myocardial oxygen deficiency, which often results in left ventricular dysfunction and contributes to HF [18]. Despite HF’s significant burden, research evaluating the efficacy of EA in enhancing cardiac functional capacity and quality of life in patients with HFrEF remains exceedingly scarce, especially for studies with human subjects and longer durations. Many previous studies have relied on animal models and brief intervention periods (often 10 sessions), highlighting the need for extended interventions in human populations.

The study aimed to address the research gap by implementing 32 therapy sessions over four months, following an initial three-month period of stable, cardiologist-led treatment. The study duration was selected to align with the extended vulnerable phase in HFrEF, which typically persists for three to six months post-discharge and necessitates intensive multidisciplinary care to reduce readmission and enhance QoL [19, 20]. Our previous studies (2023) demonstrated that two months of EA therapy (16 sessions) improved heart function, although the difference compared to drug therapy was not statistically significant [10]. Therefore, this study extended the therapy duration to four months and 32 sessions to more thoroughly assess the potential benefits of EA.

Electroacupuncture (EA) stimulates neurotransmitter systems, messenger Ribonucleic Acid (mRNA), and c-Fos expression over 30 min. This process decreases activity in parasympathetic cardiovascular neurons in the rostral ventrolateral medulla (rVLM) and the paraventricular nucleus (PVN), resulting in reduced sympathetic outflow and lower blood pressure (BP). The hypothalamus plays a mediating role by linking to brain areas such as the arcuate nucleus, ventrolateral periaqueductal grey, raphe pallidus, rVLM, and PVN, which together influence how EA lowers BP [21].

Assessing QoL in HFrEF patients is critical for understanding both physical and emotional disease impact [5, 7]. Three standard measures – LVEF, the 6MWT, and the KCCQ-23 – are used to provide complementary information [22, 23]. Together, these tools offer a comprehensive view of QoL in HFrEF patients [9, 24].

The comparison of delta LVEF measurements in the Intervention Group before and after therapy revealed an increase of 14.07 ± 5.67%, which was statistically significant (*p* < 0.05). In the Control Group, the pre-therapy and post-therapy LVEF measurements demonstrated an average increase of 6.22 ± 14.45%, which was not statistically significant (*p* > 0.05). The mean delta LVEF between the two groups was significantly different (*p* < 0.05). These findings are consistent with those of Ma et al. (2014), who assessed male rats with chronic HF treated with EA at PC5 and PC6 acupoints. Their study demonstrated significant improvement in LVEF compared to the sham group, suggesting a potential role for EA in HF treatment. The authors concluded that EA weakened RSNA and CSAR in chronic HF rats, while improving cardiac function and remodelling, which supports its potential utility in cardiology and alternative medicine [12].

The 6MWT test is simple, objective, and inexpensive, and can be completed efficiently. It is used to assess functional capacity and to help determine prognosis for patients’ daily life activities. The 6MWT has become widely used for evaluating submaximal functional exercise capacity [15, 25]. Studies have shown that the Six-Minute Walking Distance (6MWD), as measured by the 6MWT, is an established indicator of functional capacity in HF patients [26, 27]. This study identified a significant difference in the mean increase in 6MWD between the Intervention Group and Control Group (*p* < 0.05). Combination therapy of pharmacological drugs and EA increased the mean 6MWD by 348.82 ± 61.23 m over 4 months. These findings are consistent with those of Kristen et al. (2010), who reported that acupuncture improves ventilator efficiency, post-exercise recovery, and prevents skeletal muscle fatigue [28].

Carvalho et al. (2011) found that the distance in the 6MWT strongly predicts mortality and rehospitalization in HF patients [29]. Liang et al*.* (2019) reported that acupuncture and moxibustion improve 6MWT performance. According to the Texas Heart Institute, patients walking under 300 m in the 6MWT with LVEF < 30% have a much higher risk of death [9].

The delta KCCQ-23 score increase was much greater in the Intervention Group than the Control Group (34.94 ± 5.99 vs 69.71 ± 8.07; *p* < 0.05). Though prior studies did not use KCCQ-23 for acupuncture in HF, research using the SF-36 and Minnesota Living with Heart Failure Questionnaire found significant QoL improvements with acupuncture [30, 31]. These results align with earlier reports. Changes in KCCQ-23 scores correlate with changes in the 6-min walk test (6MWT). For example, Flynn et al. (2009) observed a 5-point increase in KCCQ, corresponding to a 112-m increase in 6MWD [32]. Kosiborod et al. (2007) found that a 5-point increase in the KCCQ led to a ~ 10% decrease in the risk of adverse events, and PC5 and PC6 were selected to inhibit the sympatho-excitatory reflex by activating mu and delta receptors, thereby suppressing sympathetic activity and improving cardiac function [17]. These acupoints enhance the vagal component of heart rate variability. Electroacupuncture at ST36 and SP6 restores homeostasis through opioid-mediated mechanisms, including beta-endorphins and enkephalins, which reduce inflammation and blood pressure. When blood pressure is low, stimulation of ST36 can decrease sympathetic nervous system activity and increase mean arterial pressure [33]. This coordinated approach guides the selection of acupoints for EA in HFrEF. EA at HT7 may influence autonomic heart regulation by engaging the parasympathetic system, which increases HR variability and potentially relieves myocardial ischemia injury [34]. EA at the BL14 and BL15, located above the 4–5 thoracic nerves near the dorsal ganglia and sympathetic nerves to the heart, can modulate autonomic nervous system regulation.

Impaired function in heart failure with reduced ejection fraction (HFrEF) restricts the delivery of oxygenated blood. In response, the heart activates the sympathetic nervous system (SNS) and the renin–angiotensin–aldosterone system (RAAS), which increase preload and afterload but ultimately diminish contractility and cardiac output. A reduced left ventricular ejection fraction (LVEF) results in skeletal muscle myopathy and heightened ergoreflex activity, leading to increased sympathetic drive and ventilation, and exacerbating symptoms. Clinically, these changes manifest as shortness of breath and fatigue [25]. Electroacupuncture may reduce sympathetic activity and enhance parasympathetic activity through vagal reflexes and anti-inflammatory effects.

No electroacupuncture-related side effects, including pain, infection, or hematoma at the puncture site, were observed during the study. Serious adverse events, such as anaphylactic shock and bleeding, did not occur. Study limitations include the presence of uncontrolled comorbidities, such as diabetes, hypertension, chronic obstructive pulmonary disease (COPD), heart disease, and family history, which may confound the results. The small sample size (34 participants in randomized controlled trial) and limited ability to apply strict inclusion criteria due to patient availability and time constraints are also acknowledged.

## Conclusions

This study evaluated the effect of combining pharmacological therapy with electroacupuncture on quality of life in patients with heart failure with reduced ejection fraction (HFrEF). The findings indicate that EA significantly reduces sympathetic activity and increases parasympathetic activity, as evidenced by improvements in Left Ventricular Ejection Fraction (LVEF), Six-Minute Walking Distance (6MWT), and Kansas City Cardiomyopathy Questionnaire-23 (KCCQ-23) scores. These results support the use of electroacupuncture as an adjunct to conventional treatment for optimizing quality of life in HFrEF patients.

## Data Availability

No datasets were generated or analysed during the current study.

## Abbreviations

HF: Heart Failure
CVD: Cardiovascular Disease
EA: Electroacupuncture
HFrEF: Heart Failure reduced Ejection Fraction
RCT: Randomized Controlled Trials
LVEF: Left Ventricular Ejection Fraction
6MWD: Six-Minutes Walking Distance
KCCQ-23: Kansas City Cardiomyopathy Questionnaire-23
ESC: European Society of Cardiology
ACA: American College of Cardiology
AHA: American Heart Association
EF: Ejection Fraction
CO: Cardiac Output
HPA: Hypothalamus-Pituitary-Adrenal
RSNA: Renal Sympathetic Nerve Activity
CSAR: Cardiac Sympathetic Afferent Reflex
TCM: Traditional Chinese Medicine
HT: Heart meridian
PC: Pericardium meridian
BL: Bladder meridian
ST: Stomach meridian
SP: Spleen meridian
6MWT: Six-Minutes Walking Test
QoL: Quality of Life
SNS: Sympathetic Nervous System
MAP: Mean Arterial Pressure
RAAS: Renin Angiotensin and Aldosterone System
CAD: Coronary Artery Disease
mRNA: Messenger Ribonucleic Acid
rVLM: Rostral Ventrolateral Medullar
PVN: Paraventricular Nucleus
BP: Blood Pressure
